# Racial Differences in Neighborhood Perceptions and their Influences on Physical Activity among Urban Older Women

**DOI:** 10.3934/publichealth.2017.2.149

**Published:** 2017-04-21

**Authors:** Wenjun Li, Elizabeth Procter-Gray, Gretchen A. Youssef, Scott E. Crouter, Jie Cheng, Kristen Brown, Linda Churchill, Anthony Clarke, Judith K. Ockene, Michelle F. Magee

**Affiliations:** 1 Health Statistics and Geography Lab, Division of Preventive and Behavioral Medicine, University of Massachusetts Medical School, 55 Lake Avenue North, Worcester, MA 01655, USA; 2 MedStar Diabetes and Research Institutes, 100 Irving Street, NW, East Bldg. #4114, Washington, DC 20010, USA; 3 Department of Kinesiology, Recreation and Sport Studies, The University of Tennessee Knoxville, 1914 Andy Holt Ave, Knoxville, TN 37996, USA; 4 Georgetown University School of Medicine, 3900 Reservoir Road, Washington, DC 20057, USA

**Keywords:** race, aging, neighborhood perception, physical activity, women's health

## Abstract

**Background:**

Proper levels of physical activity (PA) are important to healthy aging. Little is known about racial differences in influences of neighborhood perceptions (NP) on PA and use of neighborhood resources among community-dwelling older women.

**Materials and methods:**

In 2014 and 2015, 49 white and 44 black women of age 65 and older living in Washington, DC were queried about their PA, NP, use of neighborhood resources and sociodemographic characteristics. They wore an accelerometer and a Global Positioning System device concurrently for 7 consecutive days. Data were analyzed by race.

**Results:**

Compared to Whites, Blacks had lower NP scores (71% positive *vs.* 77%, *p* = 0.01), lower mean daily step counts (mean (SD): 3256 (1918) *vs.* 5457 (2989), *p* < 0.001), and lower frequencies of all exercise activities combined (19.7 (8.7) *vs.* 25.2 (11.8) per week, *p* = 0.01). For both Whites and Blacks, better NPs were associated with more frequent PA both at (*p* = 0.05) and away from home (*p* = 0.01). However, better NPs were associated with higher frequencies of exercise activities, moderate-to-high intensity activities, and utilitarian walking for Whites but not Blacks (*p* < 0.05 for race-perception interaction terms).

**Conclusions:**

In an urban setting, older Black women were more likely than older White women to have poor NPs, less PA, and weaker or no association of positive NPs with higher levels of certain PAs. Such substantial racial differences warrant further investigation and consideration in health promotion programs.

## 1. Introduction

Physical activity (PA) is important to healthy aging. Proper levels of PA are important for the prevention of obesity and its co-morbidities and to the maintenance of good health and independent living among older women [1]–[3]. Moderate PA, such as walking, has been widely recommended by CDC and health professionals to older adults to reduce risk for chronic diseases and disabilities [4],[5]. Benefits of regular walking in older women include reduced risk for mortality [6],[7], coronary heart diseases [8],[9], diabetes [10], hip fracture [11],[12], fewer depressive symptoms [13], better ability to maintain functional capacity [14], better cognitive function with less cognitive decline [15], and more social involvement [16]. Compared to White women, older Black women carry a disproportionally higher burden of cardiometabolic diseases [17] and related risk factors [18],[19]. Formulation of effective interventions requires a thorough understanding of the determinants of their PA behaviors including racial differences.

Although personal determinants are well documented in the literature, less is known about the influence of older adults' residential environment and their perceptions of access to and quality of their neighborhood environment on their PA. Several built environment factors have been associated with adult PA [20]–[22] including low population density in suburbs [23], motor vehicle dependency [24], traffic volume and safety [25],[26], proximity to shops and health care facilities [25], lack of access to fitness facilities [27], poor land use mix and lower “walkability” [28], and limited access to public parks and recreational areas [29]. Availability and conditions of sidewalks or walkways in neighborhoods affect the PA level of older residents [30],[31]. Ability to make utilitarian walking trips from home and perception of favorable neighborhood surroundings for walking are associated with increased PA levels in older women [32]. A particular knowledge gap is the inadequate understanding of an older adult's utilization of neighborhood resources such as frequency, location, and determinants of space and time use for physical activities. Such data can inform the design of health promotion programs targeting the specific needs of older residents and planning of age-appropriate neighborhood resources.

Older adults may have more physical limitations and no longer routinely travel outside their neighborhoods to work. Therefore, their daily living, health and well-being may depend more on neighborhood resources in close proximity to their homes. Compromised mobility and reduced income and ability to drive limit their use of fitness facilities. A good pedestrian network and public parks near home are crucial resources for them to remain active and socially connected. Therefore, access to critical resources for healthy aging may influence healthy aging in various aspects. In this study, as a component of our systematic inquiries of neighborhood environmental impact on health behaviors of older adults, we investigated differences in neighborhood perceptions (NP) and their influences on PA between White and Black older women. We hypothesized that racial differences in (1) preferences in types and locations of PA; and (2) NP may exist due to differences in health conditions, culture, social norm, education, family income and neighborhood environment. We further hypothesize that there is racial difference in the strengths of influences of NP on activity and mobility patterns. To our knowledge, such racial differences are not yet well studied. A better characterization of the differences may inform the design of racially and culturally appropriate programs that promote PA among community-living older adults.

## 2. Materials and Methods

### 2.1. Study Settings and Participant Recruitment

Ninety-seven community-dwelling White and Black/African American women, aged 65 years and older, from the Washington, DC metropolitan area were recruited for the study. The participants included 49 White, 44 Black, and 4 women of undisclosed race who were not included in this analysis. Because significant sex differences in PA behaviors are expected and research funding is limited, this study focused on older women. Participants were recruited from venues with which MedStar Washington Hospital Center has established hospital-community relationships. To increase the diversity and representativeness of the study sample, an area-based strategy was used for recruitment. We recruited (1) Black women in Black predominant neighborhoods (BPN) in recreation and senior centers, places of worship and senior housing complexes; (2) White women in BPN via neighborhood listserves, local universities, hospitals, medical practices and clinics; (3) Black women in White predominant neighborhoods (WPN) in Black sororities and professional groups, e.g. Black Nurses Association, National Medical Association; (4) White women in WPN in senior groups and residences, places of worship and medical practices. Potential recruitment sites were approached to invite buy-in to offer study participation to their members/residents. Promotional materials, interest surveys and brief presentations were offered at each venue, tailored to meet site preferences. Presentations were made in housing developments, at community, senior and recreation centers. The presentation was tailored to the audience and the time available at the site for the presentation. For example, very short presentations were made at bingo in the recreation center due to the audience and time available, whereas more time would be available at senior centers and in housing developments. At senior centers, interested participants would tell other potential participants about the project. In the housing development, a one-on-one recruitment approach was used in which residents in common areas were approached about the study. Flyers were distributed in the clinic as well as all other sites.

To be eligible for the study, an older woman had to be self-identified as Black or White, age 65 or older, English speaking, ambulatory with or without assistive devices, willing and able to perform all study-related activities independently or with a designated caregiver, and able to pass cognitive function screening on the Short Portable Mental Status Questionnaire (SPMSQ) [33] with 3 or fewer errors. Four women who expressed strong interest but were not willing to classify themselves as White or Black at the time of assessment were also included in the study but not in the analyses presented in this paper.

Once consented, each participant was offered a choice of method of participation via: small groups; one-on-one site visits with a study team member; by mail, or by telephone to facilitate individual participant preferences and needs. Survey instruments completed at home were returned by mail using a study-provided pre-paid envelope. The two batteries of instruments take about 2–3 hours to complete. The first battery included surveys of demographics, health and health care, lifestyle factors, anxiety, lower extremity problems, and fall history and efficacy. The second battery included instruments on self-report PA, general nutrition, and food purchasing habits, activity time and place, neighborhood perceptions, and depression. The questionnaires are purposefully designed and formatted for older adults, with large font typesets and high contrast printing, and in-person or telephone interviews for individuals with vision problems. In the week following the completion of the first battery, participants wore an accelerometer and GPS device for 7 consecutive days. Details about the instruments are described below.

The study protocol was approved by the MedStar Health Institute Review Board (Docket # 2014–261). All participants provided a written consent.

### 2.2. Personal Data

Each participant completed a series of questionnaires regarding her sociodemographic characteristics, physical and mental health conditions, lower extremity symptoms and problems, history of falls and fall injuries, health care utilizations in the past year, and lifestyle habits itemized in Table 1. Assessment of most characteristics was by simple self-report, e.g., “Have you ever been diagnosed or treated by a doctor for any of the following conditions…?” However, the following standardized instruments were also administered: the Tinetti Falls Efficacy Scale for fear of falling [34], Beck Anxiety Inventory [35], CES-D Depression Scale [36]. Each participant received three unannounced computer-assisted 24-hour dietary recalls (24HRs), conducted on randomly selected days within a 1-week period (two weekdays and one weekend). Diet quality was measured using the alternate healthy eating index (AHEI) [37], which had a total possible score ranging from 0 to 80 points with higher scores indicating better overall dietary quality.

**Table 1.** Characteristics of participants (Mean (SD) or percent).

	Overall (n = 97)	White (n = 49)	Black (n = 44)	*P*-value ^1^
**Sociodemographic**				
Age (years)	74.2 (7.0)	75.9 (7.3)	72.1 (5.3)	**0.01**
Education (years)	16.3 (3.0)	17.5 (2.5)	14.9 (3.1)	**<0.01**
Annual family gross income < $50,000	48.4	35.4	60.5	**0.02**
Married or living with partner	36.1	36.7	34.1	0.79
Living alone	48.5	63.3	31.8	**<0.01**
**Physical and mental health**				
CESD Depression Scale	7.7 (6.6)	7.5 (6.5)	7.6 (6.9)	0.94
Beck Anxiety Scale	5.5 (6.6)	6.3 (6.9)	4.8 (6.5)	**0.05**
Number of comorbid conditions ^2^	1.7 (1.1)	1.6 (1.2)	1.7 (1.1)	0.94
Currently taking ≥ 2 medications ^3^	39.6	37.5	43.2	0.58
Heart or circulatory condition	60.8	51.0	68.2	0.09
Diabetes	23.7	12.2	34.1	**0.01**
Respiratory disease	13.4	12.2	15.9	0.61
Cancer	4.1	6.1	2.3	0.36
Rheumatoid arthritis	5.2	2.0	9.1	0.13
Osteoarthritis	22.7	30.6	13.6	**0.05**
Osteoporosis	10.3	18.4	2.3	**0.01**
Poor vision	8.2	12.2	2.3	0.07
Physical limitations (ADL)	3.1	4.1	2.3	0.62
Hospitalized in past 3 mos.	5.2	2.0	9.1	0.13
Number of indoor falls in past 6 mos.	0.1 (0.4)	0.2 (0.4)	0.1 (0.4)	0.18
Number of outdoor falls in past 6 mos.	0.2 (0.4)	0.3 (0.4)	0.1 (0.3)	**0.01**
Tinetti Falls Efficacy Scale	15.3 (15.9)	16.4 (17.5)	12.9 (11.1)	0.24
**Lifestyle**				
Current smoker	7.2	6.1	6.8	0.89
Weekly frequency of alcohol use	1.6 (2.1)	2.6 (2.4)	0.7 (1.3)	**<0.01**
Paid or volunteer employment	15.6	16.3	16.3	1.00
Healthy Eating Index (AHEI)	38.8 (11.8)	43.9 (33.5)	33.5 (10.2)	**<0.01**
Monthly freq. of eating at restaurants	3.2 (3.9)	3.8 (3.5)	2.5 (4.3)	0.10

^1^ Chi2 test for categorical variables, and Wilcoxon rank sum test for continuous variables.

^2^ Number of comorbidities from the following list: heart or circulatory conditions (stroke, ischemic attack, high blood pressure); respiratory (asthma, COPD); cancer or malignant tumor; rheumatoid arthritis (excluding rheumatism); intestine or colon polyps or adenomas; gallbladder disease or gallstones; systemic lupus erythematosus (lupus); kidney or bladder stones (renal or urinary calculi); diabetes; cataracts; glaucoma; osteoporosis; and osteoarthritis.

^3^ Medications taken for any of the comorbid conditions listed.

### 2.3. Physical Activity

Physical activity (PA) was measured both objectively and by self-report. Objective measurement of step counts was made with an accelerometer (ActiGraph GT3X-Plus) worn by each participant during all waking hours for 7 consecutive days. A daily mean number of steps was then calculated for each person, dropping any days in which less than 10 steps were recorded (an indication that the accelerometer was not worn).

Self-reported measures of PA included (1) frequency of all exercise (including occupational as well as recreational) activities, as measured with the self-administered CHAMPS (Community Healthy Activities Model Program for Seniors) survey [38]; (2) frequency of moderate-to-high-intensity exercise, also evaluated with the CHAMPS survey; (3) frequency of performing physical activities in the home, queried as, “How often did you do physical activities in your home (for example cooking, washing, vacuuming, climbing stairs) or gardening in the last month?”; (4) frequency of performing physical activities away from home, “How often did you leave your home to do physical activities in the last month (including activities such as walking in your neighborhood, going to the grocery store)?”; (5) frequency of walking for utilitarian purposes, “How often do you walk to the store, post office, bank or other businesses in your neighborhood?”; and (6) frequency of walking for recreational purposes, “Besides walking to stores or businesses, how often do you walk for at least 10 minutes for exercise in your neighborhood?”

**Figure 1.**
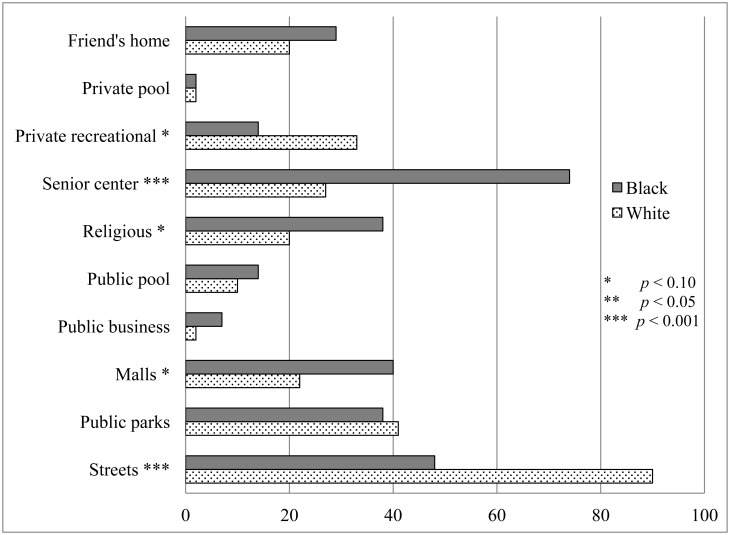
Percent of women using specified locations for physical activity at least once per month, by race.

To investigate differences in preferences for place of PA, we asked, “When performing physical activities *away from home*, do you usually do them indoors or outdoors?” Participants were also asked to choose from a list of 10 locations any at which they had performed PAs within the past month (see Figure 1).

### 2.4. Perceptions of the Neighborhood Environment

Neighborhood perceptions (NP) were queried using a structured questionnaire, REACH Study Neighborhood Perception Survey, developed by the study team. The questionnaire assesses individual perceptions of PA supports in the social and physical environment, such as “How safe do you feel out alone in your neighborhood during the day (very safe, safe, unsafe, very unsafe, don't know)?” In order to calculate aggregated perception scores, the questionnaire items were first made uniform in valence (greater value indicating a more positive perception) and in range (each item worth a maximum of 1 point). Since the item missingness was minimal (< 5%), we calculated the mean total perception score for each woman as the simple average of her non-missing perceptions. Factor analysis was then performed on all 51 perception variables to derive four domain-specific scores. The four derived NP scales were (1) safety, (2) community cohesion (social sense of community and trust), (3) accessibility (proximity to neighborhood resources), and (4) aesthetic (attractive houses and landscapes). Because factor analyses indicated relatively even factor loadings, we calculated the scores as simple averages of relevant items with equal weights. Thus each scale has a potential range of 0–1 points (i.e., 0–100%).

### 2.5. Reliability of PA and NP Instruments

The reliability of the instruments used to measure PA and NP was examined by comparing responses of subjects at two distinct time points. The mean intra-class correlation coefficient for the 7 PA items was 0.55, and for the 45 items queried concerning NP it was 0.46. We also calculated Cronbach's alpha coefficient of internal consistency for each of the 4 NP scales that we obtained by factor analysis, and these ranged from 0.75 to 0.88.

### 2.6. Statistical Analysis

Participant characteristics were summarized and compared by race (White vs. Black). Chi-squared tests (for percentages) and Wilcoxon rank-sum tests (for continuous variables) were used to test for racial differences.

White vs. Black race differences in seven PA measures of primary interest were examined using regression models. For accelerometer-measured daily mean step count and CHAMPS frequency of all exercise activities, linear regression models were used. For the other five self-report PA measures that had skewed distributions, negative binomial regression models were used. We report the coefficient obtained for race in (1) models without covariate adjustment, and (2) models adjusted for all the personal characteristics listed in Table 1. To preserve degrees of freedom in the adjusted models we derived a single composite adjustment score for each outcome for each individual using the entire set of adjusting characteristics as predictors in linear regression models [39]. Each person's single composite score used in the adjusted regression model was then predicted as the sum of all the products of each specific regression coefficient multiplied by her value of the corresponding characteristic.

Black *vs.* White differences in preferences for PA type and location were illustrated graphically using bar charts, and tested using chi-squared tests.

Mean scores for total percent positive perception and for perception scales were compared between the races using Wilcoxon rank-sum tests. We examined the associations of total NP scores with our seven measures of PA using regression models. To assess whether there were any racial differences in the associations, we included and tested interaction terms between the Black race indicator and the NP scales. We present the associations for the population as a whole as well as by race. Finally, we performed unadjusted and adjusted regressions of three PA measures, mean step count and utilitarian and recreational walking frequencies (the PAs most likely to be performed outdoors in the neighborhood), on each of the four perception scales, estimating the race-perception interaction term as well as the race-specific associations. As above, the adjusted models employed a single composite adjustment score predicted from the entire set of characteristics in Table 1. Statistical assumptions of the models, such as normality and goodness-of-fit were carefully evaluated and addressed. Potential interactions between covariables and linearity of the associations were also examined.

## 3. Results

Compared to Whites, Blacks had younger age, less education and lower household income (Table 1). Blacks were more likely to have diabetes (34% *vs.* 12%, *p* = 0.01), and less likely to have osteoarthritis (14% *vs.* 31%, *p* = 0.05) or osteoporosis (2% *vs.* 18%, *p* = 0.01). Black women were less likely to live alone (32% *vs.* 63%, *p* = 0.002), drank alcohol less frequently in the past 3 months (0.7 *vs.* 2.6 times per week, *p* < 0.001), and had somewhat lower anxiety level (mean (SD): 4.8 (6.5) *vs.* 6.3 (6.9), *p* = 0.05). Whites had more outdoor falls in the past 6 months (0.3 (0.4) *vs.* 0.1 (0.3), *p* = 0.01).

### 3.1. Level of PA

Blacks reported less activity than Whites in each of the summary measures (Table 2). The racial differences were statistically significant (*p* < 0.05) for all but PA in the home and recreational walking. After adjustment for multiple personal characteristics, racial differences in all PA measures were diminished in magnitude, but the association of Black race with lower step counts remained significant.

### 3.2. Preferred Place of PA

Among Blacks who performed PA away from home, 69% were more likely to perform PA indoors, 12% outdoors and 14% about equally indoors and outdoors. However, among White women, 29% were more likely to exercise indoors, 29% outdoors, and 41% about equally indoors and outdoors. Overall, Blacks were more likely than Whites to perform PAs indoors when exercising away from home (*p* < 0.01).

There were substantial differences in preferred locations for PA between Black and White women. Blacks were more likely to visit senior/civic centers, religious centers, and shopping malls, while Whites were more likely to exercise on streets and private recreational clubs (Figure 1). The most frequently visited PA places were senior centers for Blacks (74%) and streets (90%) for Whites.

### 3.3. Preferences in Types of PA

**Figure 2.**
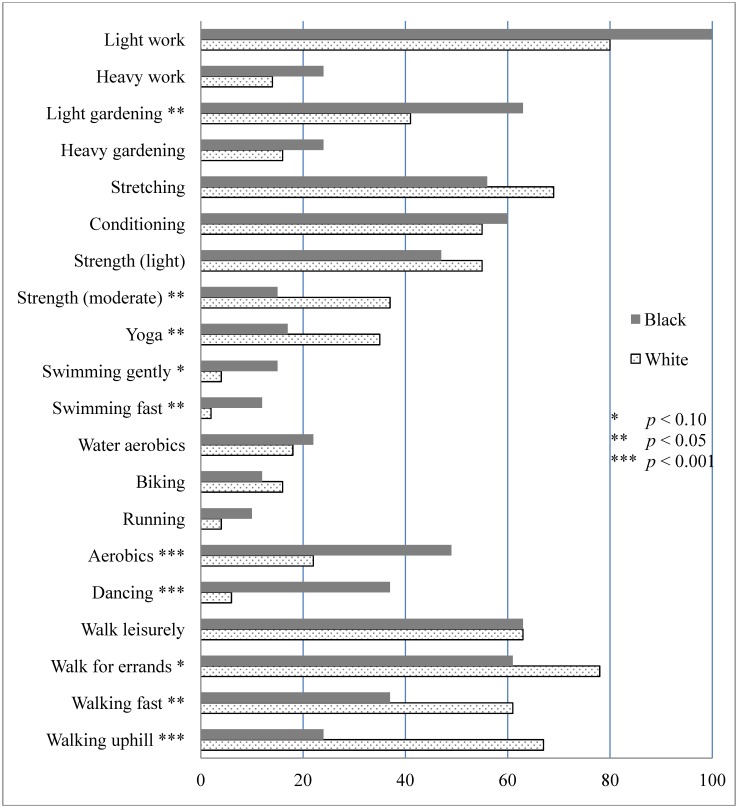
Percent of women participating in physical activity during a typical week, by type and race.

Black and White older women also had notable differences in preference for types of PA. Black women were less likely to walk fast, walk uphill, perform yoga, and do strength training, but more likely to do gardening, dance, aerobics classes, and swimming (Figure 2).

### 3.4. Neighborhood Perceptions (NP)

Compared to White women, Black women had significantly lower perceptions of their neighborhoods overall and by domain (Table 3). Blacks had a significantly lower perception of safety and of the visual attractiveness of their neighborhoods. Perceptions of the community cohesion and of the utilitarian value (accessibility) of their neighborhoods did not significantly differ by race.

### 3.5. Influences of NP on PA

Overall, more positive NP tended to be associated with higher levels of PA (Table 4). When data on Whites and Blacks were combined, there were positive associations of better NP with higher daily step counts, CHAMPS scores, frequency of PAs in and away from home, and utilitarian walking. Of these, three associations did not vary significantly by race: the associations of NP with (1) step count; (2) the frequency of doing any PA in the home (e.g., cooking, washing, vacuuming, climbing stairs or gardening); and (3) PA away from home (e.g., walking in the neighborhood, going to the grocery store). However, substantial racial differences, as indicated by the interaction term p-value in the last column of Table 4, were found in the associations of the overall NP score with several other PA measures, including CHAMPS frequency of all exercise activities, CHAMPS frequency of moderate-to-high-intensity exercise activities, and utilitarian walking frequency with (*p* < 0.05 for all). While White women had a positive association between perceptions and these PAs, Black women had a weak or even a negative association (Table 4). The racial differences were significant for the two CHAMPS scores and for utilitarian walking both before and after adjustment for personal characteristics. Although the race-perception interaction term was not significant, there also appeared to be some difference between Whites and Blacks in the impact of NP on their mean daily step counts and recreational walking habits. Only White women showed a significant positive association between perception score and step counts (Table 4).

### 3.6. Influence of Domain-Specific Perceptions on Outdoor PA

When data on Whites and Blacks were combined, there were positive associations between daily step counts and more positive perception of neighborhood safety (*p* = 0.03), community cohesion (*p* = 0.06), and accessibility (*p* = 0.06) factors in unadjusted regression models (Table 5). These associations were diminished in magnitude by adjustment for personal characteristics. Also, a higher frequency of utilitarian walking was associated with more positive perceptions of utilitarian opportunities in the neighborhood in both unadjusted and adjusted models (*p* = 0.001 and *p* = 0.04, respectively). However, safety and community perceptions had significantly different effects on utilitarian walking among White and Black women (*p* for interaction <0.03). These perceptions were associated with more utilitarian walking in White women only. Recreational walking was not associated with NP factors except in the adjusted model, where only White women reported walking more frequently when safety perceptions were higher (*p* for interaction = 0.03).

**Table 2.** Crude and adjusted differences in selected physical activity summary scores between Black and White older women.

Physical activity (PA) measure	Overall (N = 94)		White (N = 49)		Black (N = 42)		Black-White Difference **^#^**			
	Mean	SD	Mean	SD	Mean	SD	Crude	*p*-value	Adjusted **^$^**	*p*-value
Mean step count per day (x 1,000 steps)	4.46	2.80	5.46	2.99	3.26	1.92	−2.20	**<0.01**	−1.19	**<0.01**
All PA (CHAMPS, times per week)	22.4	10.9	25.2	11.8	19.7	8.7	−5.57	**0.01**	−2.77	0.17
Moderate to high intensity PA (times per week)	9.4	7.4	11.3	8.0	7.2	5.6	−0.45	**<0.01**	−-0.12	0.43
PA in home (times per month)	45.3	30.4	52.4	28.2	39.3	30.7	−0.29	0.11	−0.08	0.65
PA away from home (times per month)	30.1	24.6	37.4	26.1	22.4	18.9	−0.51	**<0.01**	−0.19	0.21
Utilitarian walking (times per month)	7.9	9.8	11.4	10.0	4.5	8.5	−0.93	**0.01**	−0.50	0.13
Recreational walking (times per month)	11	11.2	13.4	11.8	8.8	10.0	−0.42	0.18	−0.07	0.81

**^#^** We used linear regression models for the first two outcomes (step counts and CHAMPS all PA), but negative binomial models for the last five because their distributions were not normal and contained many zeros.

**^$^** Adjusted for a composite score predicted from the following characteristics: age; years of education; gross family income; marital status; living alone; alcohol consumption within past three months; smoking status; poor vision; number of indoor and outdoor falls in past six months; employment status; hospitalization within past 3 months; current heart, circulatory or respiratory disease, cancer, rheumatoid arthritis, osteoarthritis, or osteoporosis; and scores on CESD depression scale, Beck anxiety scale, and SPMSQ mental acuity scale, and Tinetti falls efficacy scale.

**Table 3.** Racial differences in neighborhood perception scales (mean ± SD), overall and by domain.

Perception score (% of responses positive)	Overall (N = 95)	White (N = 49)	Black (N = 42)	*P*-value for racial diff. **^$^**
Overall	74 ± 11	77 ± 10	71 ± 12	**0.01**
DomainsSafety	95 ± 13	99 ± 4	90 ± 17	**<0.001**
Community cohesion	69 ± 24	72 ± 23	66 ± 25	0.26
Accessibility	74 ± 29	77 ± 29	71 ± 29	0.18
Aesthetics	74 ± 20	80 ± 18	68 ± 21	**0.009**

**$** *P*-values determined by Wilcoxon rank-sum test for equality between Whites and Blacks.

**Table 4.** Associations between seven measures of physical activity and overall neighborhood perception score (range 0–1) for urban White and Black women (beta coefficients (coef.) and associated 95% confidence intervals (CI) ^$^).

	Overall			White			Black			*P* for racial diff.
Physical activity (PA) measure	Coef.	95% CI	*p*	Coef.	95% CI	*p*	Coef.	95% CI	*p*	
**I. Unadjusted**										
Mean step count per day (x1,000)	8.34	(3.07, 13.6)	**<0.01**	9.21	(0.73, 17.7)	**0.03**	1.93	(−4.23, 8.08)	0.53	0.18
All PA (CHAMPS, times/week)	28.5	(9.6, 47.5)	**<0.01**	43.4	(10.7, 76.1)	**0.01**	1.7	(−22.1, 25.5)	0.89	**0.04**
Moderate to high intensity PA (times/w)	2.25	(0.83, 3.66)	**<0.01**	3.15	(1.00, 5.30)	**<0.01**	0.22	(−1.82, 2.26)	0.83	**0.05**
PA in home (times/month)	1.54	(−0.03, 3.12)	**0.05**	0.93	(−0.97, 2.82)	0.34	1.04	(−1.59, 3.66)	0.44	0.95
PA away from home (times/m)	1.96	(0.43, 3.49)	**0.01**	0.86	(−1.34, 3.06)	0.44	1.31	(−0.71, 3.33)	0.20	0.77
Utilitarian walking (times/m)	2.57	(−0.32, 5.45)	0.08	6.97	(3.22, 10.7)	**<0.01**	−2.63	(−8.77, 3.52)	0.40	**<0.01**
Recreational walking (times/m)	1.67	(−1.08, 4.43)	0.24	2.33	(−1.46, 6.12)	0.23	−0.54	(−5.09, 4.00)	0.82	0.33
**II. Adjusted ^#^**										
Mean step count per day (x1,000)	3.38	(−0.84, 7.60)	0.12	5.58	(−0.53, 11.7)	0.07	0.15	(−5.52, 5.82)	0.96	0.09
All PA (CHAMPS, times/week)	14.0	(−3.16, 31.1)	0.11	31.01	(1.96, 60.1)	**0.04**	−4.01	(−26.1, 18.1)	0.72	**0.04**
Moderate to high intensity PA (times/w)	0.94	(−0.26, 2.15)	0.12	2.39	(0.72, 4.07)	**<0.01**	−0.45	(−2.30, 1.41)	0.64	**0.02**
PA in home (times/m)	0.49	(−0.92, 1.90)	0.50	0.59	(−1.21, 2.40)	0.52	0.23	(−2.10, 2.56)	0.84	0.95
PA away from home (times/m)	0.46	(−0.83, 1.74)	0.49	0.11	(−1.92, 2.15)	0.91	0.38	(−1.22, 1.99)	0.64	0.66
Utilitarian walking (times/m)	1.04	(−1.62, 3.70)	0.44	5.23	(1.89, 8.58)	**<0.01**	−2.04	(−7.57, 3.48)	0.47	**0.02**
Recreational walking (times/m)	1.27	(−1.07, 3.61)	0.29	2.09	(−1.60, 5.79)	0.27	0.79	(−2.69, 4.27)	0.66	0.64

**^$^** Coefficients and 95% confidence intervals estimated by linear regression for mean daily step counts and CHAMPS all PA and by negative binomial models for the others, due to their skewed distributions and large number of zeros.

**^#^** Regression models adjusted for composite score predicted from the following characteristics: age; years of education; gross family income; marital status; living alone; alcohol consumption within past three months; smoking status; poor vision; number of indoor and outdoor falls in past six months; employment status; hospitalization within past 3 months; current heart, circulatory or respiratory disease, cancer, rheumatoid arthritis, osteoarthritis, or osteoporosis; and scores on CESD depression scale, Tinetti falls efficacy scale, Beck anxiety scale, and SPMSQ mental acuity scale.

**Table 5.** Associations between three measures of physical activity and four domain-specific neighborhood perception scores (ranges 0–1) for urban White and Black women (beta coefficients (coef.) and 95% confidence intervals (CI)).

Neighborhoodperception scale	Overall			White			Black			*P* for racial diff.
	Coef.	95% CI	*p*	Coef.	95% CI	*p*	Coef.	95% CI	*p*	
**Mean daily step count (per 1000 steps)**										
Unadjusted										
Safety	5.10	(0.55, 9.65)	**0.03**	6.93	(−17.0, 30.8)	0.56	2.32	(−1.11, 5.76)	0.18	0.66
Community	2.37	(−0.07, 4.80)	0.06	2.90	(−0.79, 6.59)	0.12	0.49	(−2.01, 3.00)	0.69	0.29
Accessibility	1.94	(−0.06, 3.94)	0.06	2.70	(−0.25, 5.65)	0.07	0.44	(−1.64, 2.52)	0.67	0.22
Aesthetic	0.70	(−2.37, 3.77)	0.65	0.58	(−4.28, 5.44)	0.81	−2.03	(−5.13, 1.07)	0.19	0.38
Adjusted *										
Safety	1.70	(−1.82, 5.22)	0.34	3.48	(−13.3, 20.3)	0.68	1.15	(−2.06, 4.36)	0.47	0.64
Community	1.43	(−0.39, 3.25)	0.12	2.07	(−0.52, 4.66)	0.11	0.22	(−2.03, 2.46)	0.85	0.22
Accessibility	0.83	(−0.68, 2.34)	0.28	1.15	(−1.01, 3.31)	0.29	0.21	(−1.65, 2.08)	0.82	0.36
Aesthetic	0.91	(−1.34, 3.17)	0.42	1.61	(−1.77, 4.99)	0.34	−1.46	(−4.26, 1.35)	0.30	0.28
**Monthly utilitarian walking frequency**										
Unadjusted										
Safety	1.23	(−1.14, 3.59)	0.31	21.2	(6.03, 36.3)	**0.006**	−0.99	(−3.87, 1.89)	0.91	**0.03**
Community	1.16	(−0.38, 2.69)	0.14	2.70	(1.07, 4.32)	**0.001**	−0.20	(−0.77, 0.38)	0.50	**0.02**
Accessibility	2.12	(0.88, 3.35)	**0.001**	2.05	(0.89, 3.22)	**0.001**	1.78	(−1.00, 4.56)	0.21	0.82
Aesthetic	0.38	(−1.27, 2.04)	0.65	0.83	(−1.32, 2.98)	0.45	−1.21	(−4.30, 1.87)	0.44	0.26
Adjusted *										
Safety	-0.17	(−2.44, 2.10)	0.88	13.9	(0.50, 27.3)	**0.04**	−1.45	(−5.42, 2.53)	0.48	0.14
Community	0.45	(−0.99, 1.89)	0.54	1.98	(0.49, 3.47)	**0.01**	−0.90	(−3.81, 2.01)	0.54	0.08
Accessibility	1.21	(0.07, 2.34)	**0.04**	1.35	(0.22, 2.47)	**0.02**	1.06	(−1.41, 3.52)	0.40	0.77
Aesthetic	0.66	(−0.75, 2.06)	0.36	2.22	(0.28, 4.17)	**0.03**	−0.42	(−3.59, 2.74)	0.79	**0.03**
**Monthly recreational walking frequency**										
Unadjusted										
Safety	0.43	(−1.77, 2.62)	0.70	8.81	(−4.14, 21.8)	0.18	−0.37	(−3.38, 2.64)	0.81	0.22
Community	0.26	(−1.02, 1.54)	0.69	0.62	(−0.92, 2.16)	0.43	−-0.59	(−2.71, 1.54)	0.59	0.35
Accessibility	0.48	(−0.61, 1.58)	0.39	0.26	(−1.03, 1.55)	0.69	0.77	(−1.27, 2.82)	0.46	0.66
Aesthetic	0.48	(−1.09, 2.06)	0.55	0.68	(−1.75, 3.11)	0.58	−-0.26	(−2.64, 2.12)	0.83	0.59
Adjusted *										
Safety	0.85	(−1.25, 2.96)	0.43	15.6	(2.85, 28.3)	**0.02**	0.65	(−2.20, 3.51)	0.65	**0.03**
Community	0.95	(−0.26, 2.16)	0.12	0.81	(−0.83, 2.46)	0.33	1.62	(−0.49, 3.73)	0.13	0.79
Accessibility	0.42	(−0.58, 1.41)	0.41	0.34	(−0.82, 1.50)	0.57	0.56	(−1.29, 2.40)	0.56	0.87
Aesthetic	0.09	(−1.30, 1.47)	0.90	0.21	(−2.17, 2.60)	0.86	−0.01	(−1.95, 1.92)	0.99	0.90

Note: Coefficients and 95% confidence intervals estimated by linear regression for mean daily step counts and by negative binomial models for the others, due to their skewed distributions and large number of zeros.

* Regression models adjusted for composite score predicted from the following characteristics: age; years of education; gross family income; marital status; living alone; alcohol consumption within past three months; smoking status; poor vision; number of indoor and outdoor falls in past six months; employment status; hospitalization within past 3 months; current heart, circulatory or respiratory disease, cancer, rheumatoid arthritis, osteoarthritis, or osteoporosis; and scores on CESD depression scale, Tinetti falls efficacy scale, Beck anxiety scale, and SPMSQ mental acuity scale.

## 4. Discussion

Racial differences in levels of PA in older age have been reported in many previous studies, but remain poorly understood [40]–[42]. Previous studies focused on racial differences in amounts of PA and their influences on disease outcomes. Limited information is available on racial differences in type, location, timing and preferences of PA, and their relations with racial differences in NP. Consideration of such information is very useful when formulating racial and culturally appropriate community-based health promotion programs. This study provided new data on Black and White racial differences not only in levels but also in types and location preferences of PA, and further, novel findings on racial differences in NP and the influences of NP on PA. In densely populated urban neighborhoods in the Washington DC metropolitan area, we observed that Blacks were more likely than Whites to have poor NP and lower PA in and away from home, and association of positive NP with higher levels of certain PAs was observed only in White but not Black older women. Such detailed racial differences have not been reported in the literature, and should be further investigated to improve strategies for promoting active living in older populations.

We observed substantial differences in level, types, and preferred locations of PA between Black and White women. Consistent with several previous studies [40],[42],[43], Blacks were generally less active than Whites in each of the summary PA measures. The magnitude of these differences was mostly diminished after adjustment for multiple personal characteristics, suggesting that the differences may be related to the differences in older women's sociodemographic and health conditions. However, even after covariate adjustment, Black race remained significantly associated with lower daily step counts. Although not measured in our study, this was possibly due to the racial difference in NP, culture and residential built environment. Such determinants of PA remain to be understood.

The substantial differences in preferred locations for PA between White and Black older women are novel findings which have not been extensively examined in the past. In this study, when exercising away from home, Blacks preferred exercising indoors whereas Whites preferred outdoors. Blacks were more likely to visit senior/civic centers, religious centers, and shopping malls, while Whites were more likely to exercise on streets and in private recreational clubs. The determinants of such racial differences in location preference should be further investigated, which may shed light on effective strategies for promoting PA among older women. For example, the racial differences in income may help explain the differences in use of private recreational clubs. If Blacks had lower income and were less likely to be able to afford a private club membership, free use of public indoor places for exercise could become important to them. Many of the community-based health promotion programs emphasize the value of walking in neighborhoods and walkability of the neighborhood environment. Residents with lower income are more likely to live in low-income neighborhoods that may lack amenities and safety attributes supportive of PA [44]. Our data also suggest that the importance of public indoor spaces such as senior centers should also be considered and further evaluated when formulating health promotion programs for Black and socioeconomically disadvantaged older women.

The notable differences in preference for PA types between Black and White older women are also of programmatic importance. White women were more likely to walk fast, walk uphill, perform yoga, and do strength training while Blacks were more likely to do gardening, dance, aerobics classes, and swimming. It appeared that Blacks preferred activities in group settings and performed PAs through social events, while Whites preferred planned exercises, performing PAs alone or in small group settings. The observed differences in location preference may be attributable to the differences in preferences of PA types and settings. Consideration of such differences may make future PA promotion programs more appealing to specific racial groups.

The study also revealed substantial racial differences in neighborhood perceptions (NP) and their influences on PA. In line with previous studies [45], Black women perceived their neighborhoods more negatively, especially regarding safety and visual attractiveness, but their negative perceptions had little if any influence on their levels of PA. In contrast, White women perceived their neighborhoods more positively, and their positive perceptions were predictive of higher levels and frequency of both self-report and objectively-measured PA. Regression adjustments for personal characteristics did not alter these findings. It is unclear why such notable racial differences existed and why Black women's PA levels were not influenced by their NP. One potential explanation is that Blacks preferred exercises in group settings or social events which are likely to occur in public indoor places. The negative NP are thus less influential on indoor activities. However, one could counter-argue that the types and locations of PA preferred by Blacks may reflect their adaptations to negatively perceived neighborhoods. It is also reasonable to question why Black women did not have the same tendency to walk or exercise more where they felt safer or had more sense of community, and if it was because they had less choice about whether to walk for errands, or perhaps simply due to personal preferences. Our data, along with previous studies, highlight the importance of considering NP in studies that evaluate racial disparities in PA in relation to neighborhood social and built environments [45]. To address these issues, future studies should further examine racial differences in influences of NP on types and locations of PA, and how they are related to racial differences in personal preferences. A better understanding of these issues is of great programmatic importance.

Further, future PA promotion programs may benefit from in-depth examination of the correlations between perceived and actual built environments supportive of PA. More information is needed to understand whether the perceptions accurately reflect the objective neighborhood conditions. Because both perceived and actual neighborhood environments could become barriers or facilitators to PA [39],[45],[46], future studies should also consider objective measures of the neighborhood built environment and examine the associations between perceptions and reality, and how these two aspects may jointly influence PA among older adults.

Our study has several strengths and limitations. First, we provided novel rich data on levels, types and locations of PA of urban older women. PA was measured by self-report questionnaire and objectively by accelerometer; the two methods complemented each other. The self-report PA provided critical information on types, frequencies and location preferences of PA while accelerometers yielded objective measures of activity level. While collecting accelerometer data, we also concurrently collected PA location data using portable Global Positioning System devices, which are being analyzed to quantify location-specific PA levels. Second, our study used multiple recruitment access points and an area-based recruitment strategy to ensure the geographic, socioeconomic and racial representativeness of the participants. Third, we strategically limited the study to older women with an equal allocation of sample sizes to Blacks and Whites. Even with the relatively small sample size, the study was able to detect a number of modest to large differences that point out relatively robust racial differences in PA among older women. This cannot be accomplished without a careful sampling design. However, our study was limited in its small size, single geographic location and single sex, cross-sectional nature, and lack of observed data on actual neighborhood built environments. Although we did not intentionally exclude any woman who met the study criteria, healthier, more active and affluent women were enrolled. As a result, our results may not be generalizable to older women who are less active, homebound and of lower socioeconomic status. Except for the accelerometer data, the study relied heavily on self-report data which are subject to recall and social desirability bias. Our study protocol was well received by the participants, and the compliance rate was high which is important to further investigations. We plan to expand our studies to older men, other races/ethnicities, and other older adults living in suburban and rural neighborhoods in diverse geographic locations. Our future studies will carefully address these methodological limitations.

## 5. Conclusion

The Black to White racial differences in level, type and location of PA are substantial. Future community-based PA promotion programs should carefully consider such differences and strive to ensure that the programs meet the specific needs and preferences of older Black and White women.
